# The BDNF-Interactive Model for Sustainable Hippocampal Neurogenesis in Humans: Synergistic Effects of Environmentally-Mediated Physical Activity, Cognitive Stimulation, and Mindfulness

**DOI:** 10.3390/ijms252312924

**Published:** 2024-12-01

**Authors:** Mohamed Hesham Khalil

**Affiliations:** Department of Architecture, University of Cambridge, Cambridge CB2 1PX, UK; mhmhk2@cam.ac.uk

**Keywords:** neuroplasticity, adult hippocampal neurogenesis, dentate gyrus, brain-derived neurotrophic factor, growth factors, neurotransmitters, environmental enrichment, physical activity, cognitive stimulation, spatial complexity, mindfulness, natural environment

## Abstract

This paper bridges critical gaps through proposing a novel, environmentally mediated brain-derived neurotrophic factor (BDNF)-interactive model that promises to sustain adult hippocampal neurogenesis in humans. It explains how three environmental enrichment mechanisms (physical activity, cognitive stimulation, and mindfulness) can integratively regulate BDNF and other growth factors and neurotransmitters to support neurogenesis at various stages, and how those mechanisms can be promoted by the physical environment. The approach enables the isolation of specific environmental factors and their molecular effects to promote sustainable BDNF regulation by testing the environment’s ability to increase BDNF immediately or shortly before it is consumed for muscle repair or brain update. This model offers a novel, feasible method to research environment enrichment and neurogenesis dynamics in real-world human contexts at the immediate molecular level, overcoming the confounds of complex environment settings and challenges of long-term exposure and structural plasticity changes. The model promises to advance understanding of environmental influences on the hippocampus to enhance brain health and cognition. This work bridges fundamental gaps in methodology and knowledge to facilitate more research on the enrichment–neuroplasticity interplay for humans without methodological limitations.

## 1. Introduction

In adult humans, arguably around 700 new neurons are added in each hippocampus per day until the tenth decade of life in a process known as neurogenesis that takes place in the hippocampal dentate gyrus [1,2,3], but that rate declines not only with aging, but also through the environment, which may not be sufficiently enriched [4,5]. Nonetheless, the sustainability of this cycle is subject to various physical environment variables that have been elementally identified for the purpose of neurosustainability [6], which is a novel theory on sustaining neuroplasticity through the environment, which supports the recent interest in environmental neuroscience to explore the physical environment ingredients essential for mental health [7]. This is because adult hippocampal neurogenesis (AHN), mental health, and environmental enrichment are highly correlated concepts [8,9,10].

Research on environmental enrichment necessary for hippocampal neurogenesis and the sustainability of this process faces two significant challenges: the overly complex environment with overlapped and confounding variables [11] and the methods used for humans, which are either postmortem or involve brain imaging for hippocampal volume changes [12,13,14]. The hippocampal volume can be interpreted in multiple ways, as it is reduced in the case of psychiatric conditions, such as depression [15,16], post-traumatic stress disorder (PTSD) [17], and schizophrenia [18], or in the case of cognitive function such as late-life cognitive decline [19] and cases of mild cognitive impairment and Alzheimer’s disease [20]. This problem may inhibit the untangling of the overly complex real-world environment, isolating variables in that environment and observing changes in hippocampal volume. Total hippocampal volume changes may differ from increased subiculum volume or the dentate gyrus. Since neurogenesis is an ongoing cycle associated with the dentate gyrus [21], tracing an increase in dentate gyrus or total hippocampal volume post-synaptic integration would be highly challenging without understanding all environmental variables. Such challenges limit the interrelationship between environmental enrichment and neurogenesis in humans who increasingly suffer from mental health disorders, chronic stress, and poor cognition [22,23], indicating an unsustainable hippocampal neurogenesis cycle or at least a lack of neurogenesis stimulation to counterbalance the effects.

To date, AHN in humans still faces problems, mostly challenges and limitations of using immunohistochemistry [24]. Recently, researchers have put effort into overcoming this gap between rodents and humans by relying on the brain-derived neurotrophic factor (BDNF) in quantifying environmental affordance for physical activity for hippocampal neurogenesis sustainability in healthy subjects [25] and determining the relationship between the level of environmental enrichment and the brain-derived neurotrophic factor (BDNF) in subjects with major depressive disorder [26]. However, this paper argues that BDNF responds to a complex interplay of enrichment mechanisms that need to be understood for higher optimization of neurotrophic factor regulation.

In that regard, this article maps the path between environmental enrichment and hippocampal neurogenesis, each deconstructed into mechanisms with BDNF-interactive pathways for hippocampal neurosustainability and immediate assessment of environmental enrichment. BDNF is known for its immediate and short-term changes, which are also known to be directly associated with neuroplasticity and mental health [27,28] through working synchronously with other growth factors and neurotransmitters. The model in this paper promises to bridge a critical gap in environmental enrichment research for humans.

## 2. The Triple-Pathway BDNF-Interactive Model

**Figure 1:**
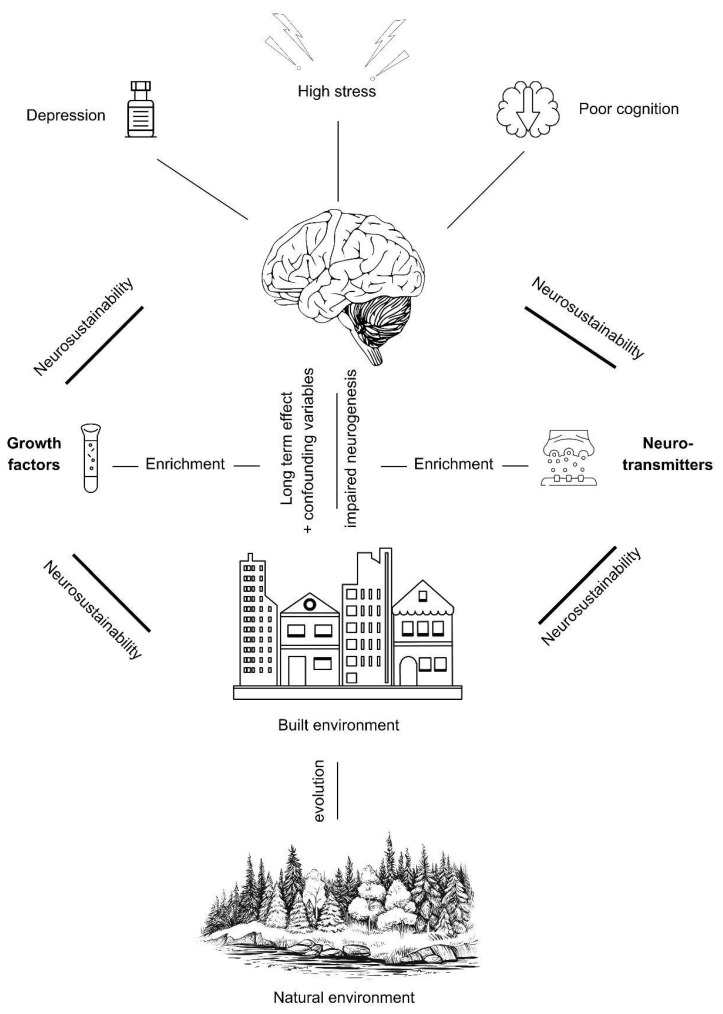
A conceptual molecularly mediated model for hippocampal neurosustainability.

At the conceptual level, as illustrated in Figure 1, the model is developed considering the effectiveness of hippocampal neurosustainability of the neurogenesis cycle that is hypothesized to take place through growth factors (including BDNF) and other neurotransmitters at the molecular level before structural changes take place.

**Figure 2:**
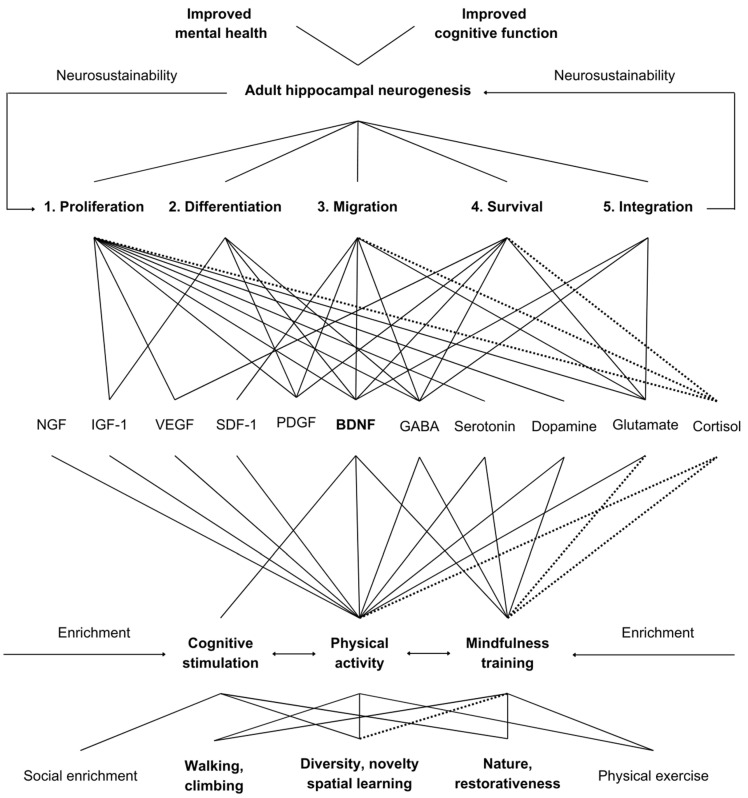
The path between environmental enrichment and hippocampal neurosustainability in humans, where solid lines represent linear and dotted lines inverse relationships.

Figure 2 illustrates the theoretically deconstructed mechanisms through which hippocampal neurosustainability can be workable. Subsections continue to explain the complex interactions between the five AHN phases and the various molecular mechanisms, followed by the central role played by BDNF, the complex interplay between BDNF and three main environmental enrichment mechanisms (physical activity, cognitive stimulation and mindfulness), the application of those environmental enrichment mechanisms in the physical environment, and, lastly, concluding with a workable model with remarks on methodological feasibility.

### 2.1. Adult Hippocampal Neurogenesis (AHN) and Neurosustainability Through Growth Factors and Neurotransmitters

AHN is a dynamic process that can be deconstructed into five main stages: proliferation, differentiation, migration, survival, and synaptic integration [29,30]. A clear breakdown of AHN into these stages is essential for the subsequent development of a model for sustainable enrichment rather than single, intermittent, or infrequent enrichment, which may not sustain AHN’s sequential and repetitive process.

Proliferation, the initial stage, involves the division of neural progenitor cells (NPCs) residing in the subgranular zone (SGZ) of the dentate gyrus [31,32]. Proliferation increases the potential of new neurons through the increase in the number of NPCs through dividing and multiplying. NPCs are not neurons yet, but they have the potential to become them. Environmental enrichment can enhance proliferation [33,34], while chronic stress and aging can suppress this crucial initial step in AHN [35,36,37]. Key growth factors like BDNF and insulin-like growth factor 1 (GF-1) directly stimulate NPC proliferation [38,39]. Additionally, vascular endothelial growth factor (VEGF) has a primary role in cell proliferation due to its function in angiogenesis (the formation of new blood vessels) [40], where an increased blood vessel density in the hippocampus provides better oxygen and nutrient supply to support the proliferation of NPCs [34]. Neurotransmitters like serotonin generally enhance proliferation [41]. Dopamine’s effect on proliferation can be more complex, sometimes stimulating and sometimes inhibiting, depending on the specific receptor involved [42,43]. Similarly, GABA receptors may suppress or promote cell proliferation [44,45]. Platelet-derived growth factor (PDGF) is involved in proliferation and subsequent stages [46].

Differentiation and migration are sometimes recognized as one phase, but they are explored separately in this article. Differentiation is when the new cells begin specializing as they progressively acquire neuronal characteristics, developing dendrites and axons—the structures essential for receiving and transmitting neural signals, respectively [47,48]. BDNF promotes the differentiation of adult-born neurons [49], which has been reported in some studies through GABAergic transmission [50]. IGF-1 also plays an important role in differentiation after proliferation [39]. Once the immature neurons differentiate, they migrate a short distance from SGZ into the granule cell layer (GCL) of the dentate gyrus, which is their designated location within the hippocampal architecture. This targeted movement is facilitated by chemical signals, such as storm cell-derived factor 1 (SDF-1) and BDNF, which guide the neurons to their correct positions [51,52,53]. Inflammation and stress may disrupt migration [54].

Not all newly generated neurons survive, so the sustainability of the fourth phase of AHN, survival, is equally important. Only a subset of the immature neurons successfully integrate into the existing hippocampal network, while others undergo apoptosis [30]. This selective survival process is highly dependent on integrating the newborn neurons into functional circuits and the reception of appropriate trophic support. Growth factors, particularly BDNF [38,55], are essential for promoting neuronal survival. Neurotransmitters, such as glutamate and GABA, also influence not only neuronal proliferation and migration, but also survival [56,57]. VEGF interacts with BDNF at this stage to contribute to neuronal survival [58].

The final stage of AHN involves the functional integration of the surviving newborn neurons into the existing hippocampal circuitry. This process requires the formation of new synapses with both pre-existing and newly generated neurons. BDNF is also well-known for its effective promotion of synaptic plasticity [59,60]. Glutamate, the primary excitatory neurotransmitter in the hippocampus, plays a crucial role in synapse formation and plasticity [61], including long-term potentiation (LTP), a key mechanism for strengthening synaptic connections. GABA regulates synaptic integration of newly generated neurons [62].

Knowing these mechanisms is important before understanding which environmental enrichment mechanisms are evidenced to drive some or more growth factors and neurotransmitters. However, and while this paper expects a shortage of exhaustive literature, the following section explores the interrelated role of BDNF with other molecular mechanisms and its central role in neuroplasticity beyond the hippocampus before exploring the environmental enrichment mechanisms and their association with BDNF or other growth factors and neurotransmitters.

### 2.2. The Interactive Role of BDNF with Other Growth Factors and Neurotransmitters for Plasticity and Beyond the Hippocampus

BDNF, a key player in the nervous system, extends its influence beyond the hippocampus. Its intricate interplay with various growth factors, neurotransmitters, and mental health disorders underscores its pivotal role in developing a viable method despite the scarcity of human-based evidence. This potential for further research in the realm of mental health is both intriguing and motivating.

The release of BDNF is evidenced to take place in both the peripheral nervous system (PNS) and the central nervous system (CNS) [63,64], and is interchangeable. In the CNS, BDNF is expressed mainly in the hippocampus, amygdala, cerebellum, and cerebral cortex in both rodents and humans, with the highest levels found in the hippocampus [65]. Still, BDNF is transported across the blood–brain barrier through both saturable and non-saturable transport mechanisms and in a specific manner, but mainly within one hour of its stimulation [66,67], which we urge to become an effective regulation for neurosustainability. Either way, it is feasible to test changes in BDNF release in serum or plasma, with the concentrations in the former about 100-fold higher than the latter [68].

**Figure 3:**
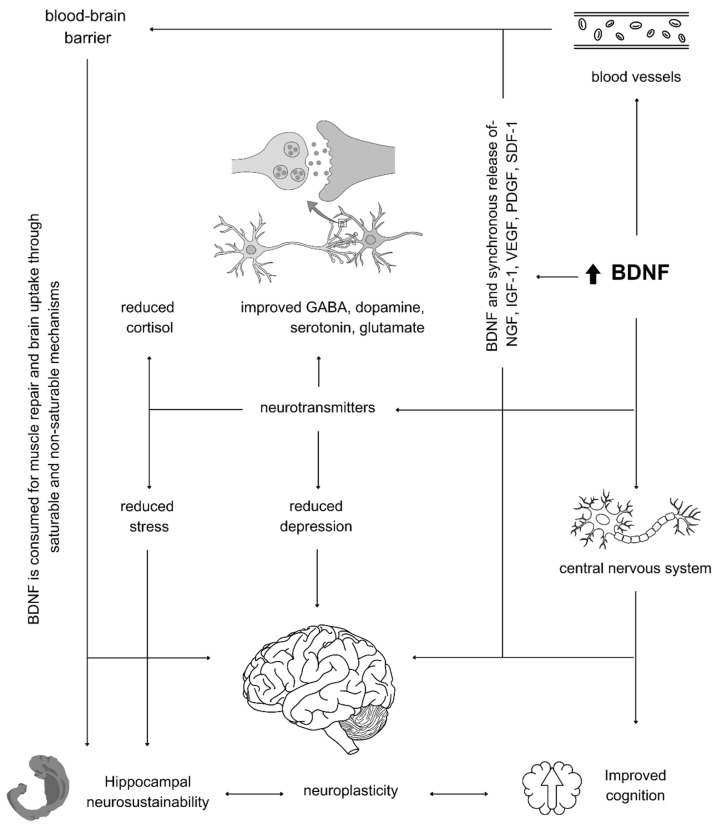
The role of BDNF with neurotransmitters in mediating depression, hippocampal neurogenesis and neurosustainability, and cerebral neuroplasticity.

The release of BDNF interacts with neurotransmitters in a complex way. For instance, the effects of BDNF on dopaminergic, serotonergic, and GABAergic neurons have been discussed [69]. BDNF may accelerate the uptake of GABA, allowing for the replenishment of neuronal pools of GABA, and BDNF has been found to enhance GABA transport in the rat cortex [70,71]. The interaction between BDNF and serotonin towards neurogenesis and synaptic plasticity was discussed by [72]. Therefore, BDNF is a crucial agent for neurogenesis and synaptic plasticity and, unsurprisingly, is reported to be associated with changes in anxiety and depression [73,74]. These complex interactions are illustrated in Figure 3.

Furthermore, the molecular mechanisms of BDNF, NGF, IGF-1, VEGF, PDGF, and SDF-1 are interrelated, often synergistically, to promote neurogenesis, neuronal survival, and synaptic plasticity. These growth factors and neurotrophic factors engage in complex signaling pathways. For instance, BDNF and NGF, since they belong to the same neurotrophin family, both share similar signaling pathways, primarily through the activation of Trk receptors (TrkB for BDNF and Trka for NGF) [75]. The activation of these receptors activates downstream signaling pathways, such as the PI3K/Akt and MAPK/ERK pathways, which promote neuronal survival, differentiation, and synaptic plasticity [25,76].

The relationship between BDNF and the other growth factors is bidirectional. Firstly, IGF-1 increases BDNF responsiveness [77], while both are known to increase signaling through the same pathway [78]. Secondly, BDNF promotes the expression and secretion of VEGF [79], while VEGF guarantees optimal blood and glucose supply to the brain while promoting the release of BDNF, and together, they improve cognitive function [80,81,82]. Both BDNF and VEGF also activate similar signaling pathways necessary for the neuroplasticity processes [83,84]. Thirdly, PDGF is found to stimulate the expression of BDNF within 2 h in the rat hippocampus [85], while both PDGF and BDNF activate similar signaling pathways such as the P13K/AKT [86]. While this is not an exhaustive list of growth factors and their bidirectional interactions, and despite that, some evidence is derived from studies conducted on mice, the release of growth factors is often interrelated, and focusing on BDNF as a critical mediating factor that is well-studied in humans can foster the testing of environmental enrichment for humans without waiting to bridge other gaps first.

### 2.3. BDNF Interactions with Three Environmental Enrichment Mechanisms: Physical Activity, Cognitive Stimulation, and Mindfulness

Environmental enrichment also holds mechanisms that this article explores and associates with similarly deconstructed mechanisms of neurogenesis in order to solidify the path for hippocampal neurosustainability. Mechanisms of environmental enrichment are categorized as physical activity, cognitive stimulation, and mindfulness, mediating different physical environment ingredients of hippocampal neurosustainability, which will be explored shortly, through the molecular mechanisms introduced earlier.

**Figure 4:**
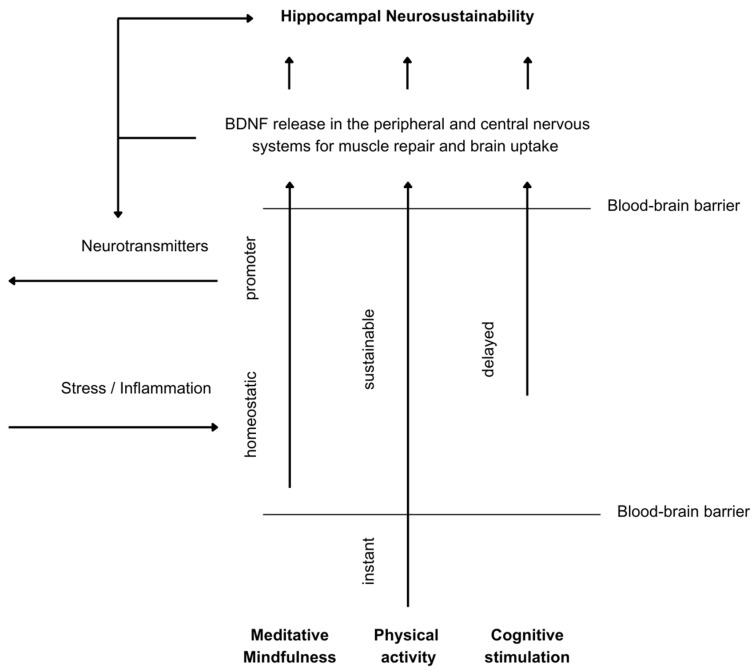
BDNF dynamics with cognitive stimulation, physical activity, and mindfulness.

The distinction of environmental enrichment into those three mechanisms can be explained through a number of recent studies exploring the different effects of physical exercise, cognitive stimulation, and mindfulness on BDNF variations. For instance, [87] explored the BDNF response in healthy older persons in response to 35 min of physical exercise, cognitive training, and mindfulness, finding that the former increased significantly, noticeably due to the rapid increase in the peripheral nervous system. But that increase lasted for one hour, which we validate here as sufficient for permeability through the blood–brain and blood–nerve barriers [66,67]. Contrary to a single 35 min intervention, [88] extended the study to 35 min/day, 5 days per week for 5 weeks, finding that only the group that underwent cognitive training increased their serum BDNF levels after 5 weeks of training. Those studies explain the nuanced differences in the molecular mechanisms’ responses to various environmental enrichment properties. Still, physical exercise is reported in some studies to have long-term effects on BDNF depending on variability [89], which will be explored in subsequent sections. Regarding mindfulness, it is argued that its effect may lie in a reduction in systemic inflammation to help reduce BDNF eradication [90,91], which shows that environmental enrichment mechanisms have integrative rather than parallel effects on BDNF release, contributing towards hippocampal neurosustainability. The integrative dynamics of the three environmental enrichment mechanisms of BDNF are illustrated in Figure 4.

With BDNF having different dynamics with cognitive training, mindfulness, and physical activity, the latter itself has different interrelationships with the different growth factors and neurotransmitters compared to its relationship with BDNF. The variables explored regarding physical activity are single bouts of physical activity, short-term physical activity, long-term physical activity, and the intensity and type of physical activity. On the one hand, for growth factors, BDNF, IGF-1, and VEGF are most explored, while NGF and SDF-1 are least explored. Firstly, BDNF is responsive to a single session of exercise, while regular exercise intensifies the effect of that single bout [92,93,94]. The intensity of physical activity increases BDNF concentration, and that response is influenced by the physical fitness level [95]. However, the specific physical activity type and dose for optimal BDNF release were not clear until recently [96], when it was identified that high-intensity interval training best modulates the BDNF levels in adults and even has potential long-term effects [97]. This is in line with an earlier review by [98], except that the latter review identified that intensity training is the only type of physical activity that is more effective than high-intensity interval training. Secondly, while also part of the neurotrophins family along with BDNF, NGF is not widely explored in humans in response to physical activity except that it has a positive relationship with long-term training among elderly women [99]. Thirdly, regarding IGF-1, disparities in the type of physical exercise, protocols, and samples under different conditions have been found in a systematic review, hindering the establishment of a consensus on IGF-1, physical exercise, and cognition [100]. A later review confirmed the latter conclusions, finding that IGF-1 serum concentrations are altered by exercise type, but in conditions that are not well-defined [101]. Last but not least, SDF-1 is found to be upregulated by aerobic exercise, while VEGF-A is upregulated by both aerobic and anaerobic types of exercise among the elderly [102]. These studies explain the nuanced differences in growth factors’ responses to variability in physical activity, and while more research is needed for some, BDNF stands out as the most reliable growth factor and is associated with most stages of AHN, as introduced earlier. On the other hand, for neurotransmitters, GABA levels increase by an average of 20% after a single bout of high-intensity interval training [103,104]. A recent systematic review found that GABA increases after acute physical activity, while studies on glutamate are inconclusive [105]. As for dopamine and serotonin, it was found in a study on patients with Parkinson’s disease that a two-week aerobic exercise mode induced distinct beneficial effects on neurotransmitter concentrations, including dopamine and serotonin, showing significant short-term effects [106]. The findings from the latter study can further support a recent review identifying that physical exercise has robust effects on dopamine [107], while intensive acute exercise increases serotonin [108]. It appears that the growth factors and neurotransmitters may respond to a similar extent to physical activity characteristics such as high-intensity interval training, which yields immediate and sustainable results. Still, more research is needed to explore specific molecular mechanisms where gaps are identified. Regulation of cortisol levels is improved through physical activity [109], and the association between mindfulness and the facilitation of BDNF release mentioned previously is also mediated by the reduction in stress and cortisol [110], which further highlights the additive effect of an integrative activity–mindfulness approach, as explored later in this section.

Cognitive stimulation has a potentially long-term additive effect on the release of BDNF, which is central to most of the neurogenic and neuroplasticity functions, while the relationship between cognitive stimulation and the other growth factors and neurotransmitters remains largely unexplored in humans. Refs. [88,111] show that a 5-week intervention of working memory/cognitive training increased BDNF and not physical activity or meditation. A recent study by [112] showed that resistance training combined with cognitive tasks (for 16 weeks) yielded exclusive improvements in BDNF compared to resistance training alone, which further supports the additive effect of cognitive stimulation on the release of BDNF along with physical activity. The effect size of physical activity and cognitive stimulation on the neurogenic process through the modulation of BDNF needs more research. In other words, the feasibility of physical activity may not yield a substitution to the delayed effect and molecularly niched effect of cognitive stimulation until more research explains the complex interactions.

Mindfulness training provides the needed internal environment for the release and modulation of BDNF, arguably through the prevention of systemic inflammation, while it works on enhancing the processes of the above-mentioned neurotransmitters. On the one hand, mindfulness training through meditation or exercise has been proven to have a positive relationship with BDNF. In their systematic review and meta-analysis of controlled trials, ref. [90] concluded through eleven studies that mindfulness-based interventions (MBI) can increase peripheral BDNF with both types: exercise-MBI and meditation-MBI. It is worth noting that the included studies lasted between 5 and 24 weeks. Later, ref. [113] revealed through a study that even an intensive, short period of MBI (8 h) may increase serum BDNF and reduce anxiety more than relaxation on-site, while there was no significant difference for cortisol, which we consider to warrant future research. We should not assume a correlation between the two molecular mechanisms except in certain conditions, such as, for instance, in the study cited in [114]. A recent study explored the dose–response effect of mindfulness-based therapy for anxiety and depression and the increase in BDNF [115], which this paper views as associated with neurogenesis. On the other hand, mindfulness is related to a number of neurotransmitters. Meditation processes are linked to GABA levels [116,117]. Long-term meditators have been reported to have elevated serotonin levels [118], while meditation has been found to increase dopamine levels as well [119,120], and brief mindfulness also increases glutamate metabolism [121]. On the contrary, meditation interventions efficiently reduce cortisol levels [122]; the reduction in cortisol was found in another study to be associated with improvements in serum BDNF levels and depression scores through yoga, which is suggested to be a facilitator of neuroplasticity [123]. A 3-month yoga–mindfulness retreat was associated with an increase in the plasma levels of BDNF and anti-inflammatory cytokine IL-10 [124], which shows the multifold benefits of mindfulness on the facilitation and stimulation of BDNF release through the reduction in cortisol and inflammation. However, mindfulness has mediating effects on BDNF function and the hippocampus, but does not directly correlate with hippocampal volume [125].

### 2.4. Towards Affordance of Physical Activity, Cognitive Stimulation, and Mindfulness Through the Built Environment

The real challenge begins at this stage after understanding the path to neurosustainability by regulating AHN through environmental enrichment mechanisms, which may not be well afforded by the modern built environment at its various scales, where sedentary behaviors, depression, anxiety, and poor cognition are common. This urges the exploration of means to promote physical activity, cognitive stimulation, and mindfulness through the built environment, knowing their complex interrelationships, which were introduced earlier.

Here, we first challenge the translation of environmental enrichment paradigms from rodents to humans so that we can seek appropriate means for environmental affordance of physical activity, cognitive stimulation, and mindfulness. The first criticism concerns the translation of running wheels into physical exercise interventions rather than any means of environmentally driven physical activity. The latter approach would be more beneficial [25], knowing that humans have an abundance of space to use compared to a rodent in a cage. However, the common trend is sedentary behaviors in enclosed built environments, while physical exercise is merely an antidote to part of a larger problem. The built environment has also transformed into something that does not promote mindfulness, which is known to be associated with amygdala reactivity [126], as grey spaces and built environments have adverse influences on the amygdala, which is associated with emotional processing along with the hippocampus [127,128,129,130]. Cognitive stimulation depends entirely on lifestyle patterns [131], and screen use has adverse effects [132]. These examples show the missed potential that the built environment could hold if it were designed with appropriate means of enrichment to which humans are exposed consistently and repeatedly. It can hinder neurogenesis or promote neurosustainability of the hippocampal plasticity cycle.

Physical activity, as established in the earlier discussion in this paper, is ideal in its high-intensity interval training form, yet this is not the only means of effective physical activity or the only feasibly translatable one, depending on the macro- or micro-built environment scale. For instance, on the one hand, at the macro-environment scale, walking can be an effective form of physical activity, even in its low-intensity form, as is evident in multiple studies. One study reported that a greater amount, duration, and frequency of total daily walking activity were each associated with larger hippocampal volume, suggesting that increasing physical activity may produce measurable cognitive benefits to the hippocampus through molecular pathways [133] which are mapped in this paper. Another recent study reported that free-living physical activity is associated with a larger hippocampal volume, specifically the left parahippocampal gyrus and right hippocampus, as well as greater functional connectivity in healthy older adults [134], suggesting that the macro-scale of the built environment is capable of promoting physical activity to an extent. On the other hand, at the micro-built environment scale, the challenge becomes more difficult knowing the limited space of the built environment, particularly for houses and workplaces, while for museums or commercial malls, for instance, there might be a greater chance of walkability. However, the challenge remains, as people spend most of their time either at home or work, which challenges the design of those spaces. In this regard, this paper suggests reliance on high-intensity interval training, which can be facilitated by reliance on staircases as a form of structural environmental enrichment. This interrelationship was explored by [135] in the workplace. The authors aimed to determine the receptivity to incorporating stair-climbing as an “exercise snack” in the form of three isolated bouts of ascending 53–60 stairs sporadically throughout the day. This was compared to more traditional high-intensity interval training (three bouts of 53–60 stairs within a structural high-intensity interval training workout), and it was found that the former approach has the potential to impact sedentary behaviors and physical activity metrics positively. However, it remains unclear how the physical environment, after becoming conducive to physical activity, can promote an active lifestyle in that environment through its design parameters at both the macro- and micro-scales, which is critical to consider and explore in future research using BDNF and other growth factors as indicators for environmental affordance for physical activity rather than a lifestyle-based increase in hippocampal volume.

Cognitive stimulation and training are challenging to translate into or through the built environment due to its multidimensionality of mechanisms and mediums. Nonetheless, each mechanism can have various implications. In addition to all this complexity, we explained earlier that cognitive stimulation has a delayed effect at the molecular level. In order to facilitate the affordance of cognitive stimulation, it is essential to explore how cognitive stimulation programs work. A review by [136] found that studies using cognitive stimulation programs mainly target training of memory, attention, and executive functions. Thus, translating cognitive stimulation programs through the built environment is outlined in various forms.

Firstly, cognitive stimulation needs to be better investigated through the impact of diverse activities on the different molecular mechanisms mediating the different hippocampal neurogenesis phases for neurosustainability. Research supports the notion that that greater engagement in a range of daily activities is associated with better cognitive abilities as well as a greater average hippocampal volume across the left and right hemispheres only after an 8-day period [131,137], explaining that activity diversity is a marker of exposure to and engagement with novel experiences and environments. What matters most is diversity in a short period, such as having more diverse experiences when visiting diverse locations compared to merely visiting more diverse locations in general [138]. The problem is that hippocampal neurogenesis and neurosustainability are difficult to explore after short-term exposure, which urges us to take this approach a step further and explore long-term structural plasticity changes as well as short-term molecular responses to diversity and novelty in a synergistic manner. On the other hand, novelty and diversity were explored in a recent review as a means of changing the interior spatial complexity in rodents’ housing [139], showing that it has an additive effect on neurogenesis besides physical activity and might be translatable to human environments. We can understand that the complex diversity induced by novelty is effective, but its implications between rodents and humans are different. For rodents, it implies changing the environment, while humans can experience novelty by increasing the diversity of their activities, which might still be influenced by the spatial design of the environment due to humans’ higher cognitive abilities. It is important to note that the diversity of activities includes social interaction, which is reported to be a viable lifestyle factor for preserving cognitive function [140]; therefore, social enrichment may have an additive effect.

Secondly, several studies conducted on humans have proven that navigational complexity, novelty, and spatial exploration are forms of learning through space that have positive outcomes on the hippocampus. Before delving into the details, it is essential to note that these forms of learning indeed implicitly target the training of memory, attention, and executive function. The first example is a series of studies performed on London taxi drivers through which navigation-related structural changes in the hippocampus were reported [141,142,143], interestingly revealing an impact of cognitive stimulation through navigation with physical activity not acting as a confounding variable, further cementing the argument presented in this paper. Another study where participants experienced spatial navigation training virtually while walking on a treadmill every other day over 4 months showed that this form of training modifies hippocampal volumes, confirming that mental stimulation may have direct effects on neural integrity [144]. Recently, a study by [145] showed that greater environmental complexity was selectively associated with larger allocentric, but not egocentric, navigation-related brain volumes, as well as lower rates of diagnosis of mild cognitive impairment (MCI) and Alzheimer’s disease (AD). These studies support the model developed in this paper. However, it is still difficult to tell whether the neurogenesis cycle is neuro-sustainable and whether the cycle is effectively regulated and maintained in a flow, which makes it critically important to rely on testing the mediating molecular mechanisms. It is essential to consider the variability of exposure effects between physical activity and cognitive stimulation when conducting research in real-world settings and testing molecular mechanisms afterward. Confounding variability can occur when participants are unfamiliar with the environment, for instance. Spatial exploration and route learning may occur, which, as cognitive stimulation methods, affect the molecular mechanisms, yet with a delayed effect. However, the novelty of the experience itself can lead to increased walking or physical activity that may not happen after one becomes familiar with the environment. Hence, built environments, especially at the interior level, may severely lack cognitively demanding spatial experiences and contribute to a lack of physical activity simultaneously due to mental investment in cognitively loading work or digital device use, as well as sedentary behaviors. Higher exposure to traditional forms of screen time has been found to be independently associated with lower serum BDNF levels [132], urging the built environment not only to promote physical activity, but also to contribute to additive cognitive stimulation through the spatial experience.

Thirdly, visual tasks as a means of cognitive stimulation might be most feasible for interior built environments. They are still proposed in line with the essence of diversity by targeting the training of memory, attention, and executive functions, potentially through novelty and diversity. In that regard, few studies support the role of artwork as a cognitive stimulator. Ref. [146] explored it as a method of effective cognitive stimulation for people with dementia. Ref. [147] discussed the feasibility of a six-week cognitively stimulating program focusing on the visual arts of renowned artists along with other interventions, which we see as effective for the suggested 5-week exposure to test a change in BDNF levels, suggesting that it can be an effective means of affordable cognitive stimulation at various built environment scales and building types. This approach is known as “aesthetic cognitivism”, which was proposed by [148] to refer to the ongoing thoughts, feelings, and actions of a person in response to viewing an artwork that is necessary to acquire new knowledge and understanding simultaneously with motivational states associated with learning. Thus, we propose that it can be effective for both releasing BDNF and facilitating reward-related neurotransmitters to promote neurosustainability integratively.

Last but not least, a recent systematic review found that olfactory training is effective for improved global cognition and increased volumes of olfactory-related brain regions, including not only the olfactory bulb, but, interestingly, the hippocampus as well [149]. It is worth noting that olfactory enrichment improved memory through novelty [150], which emerges again as an effective facilitator for cognitive stimulation regardless of the medium.

Regarding mindfulness, it is essential to note that exposure to nature is multifold in that it can promote both cognitive stimulation and mindfulness, and this relationship is often mediated by the built environment, with tendencies to foster state mindfulness [151]. The built environment can either promote means of communication with nature or embrace biophilia, which is arguably related to nature. The problem remains that nature is filled with intriguing stimuli that grab one’s attention in a bottom-up means, allowing top-down direction–attention abilities a chance to replenish [152]. This is opposite to urban environments filled with stimulation that captures one’s attention dramatically, but in a way that is not restorative (such as avoidance of being hit by a car), which we argue here may hinder the potential for promoting the release of BDNF and other neurotransmitters. Hence, the dynamics between cognitive stimulation and mindfulness can not only confound, but hinder each other in certain conditions in the built environment.

A plethora of recent studies have validated that exposure to natural environments has more positive impacts on neuroplasticity than urban environments [129,130]. This becomes critical at more complex levels than designing a greener urban environment, because increasing layout complexity, as explained earlier, should be cautiously explored in noisy environments, since they can mitigate stress in different ways [153]. We assert this as a hindrance to the neurosustainability process through the seamless stimulation of BDNF via other molecular mechanisms. More research is needed to explore the effects of various means of natural enrichment on increasing or merely facilitating the release of BDNF through the built environment at its various scales. Exploring the different means of integration of biophilia, which is argued to be a means for healthy dwelling, preventing depression, and improving wellbeing [154,155], is critically needed to test the biophilia approach’s potential for neurosustainability. This is in conjunction with the effects of negative inflammatory or positive cognitively stimulating factors in the built environment on the release of BDNF and other molecular mechanisms after at least 5 weeks of exposure [90]. Here, we propose that the biophilic quality can reduce the effect of stress, while its restorative effect, only when mediated with its dynamic essence of change, can be an effective promoter of cognitive stimulation for an additive effect on BDNF. Lastly, it is crucial to consider that mindfulness, like cognitive stimulation, can be confounding with physical activity if accompanied by walking [129].

**Figure 5:**
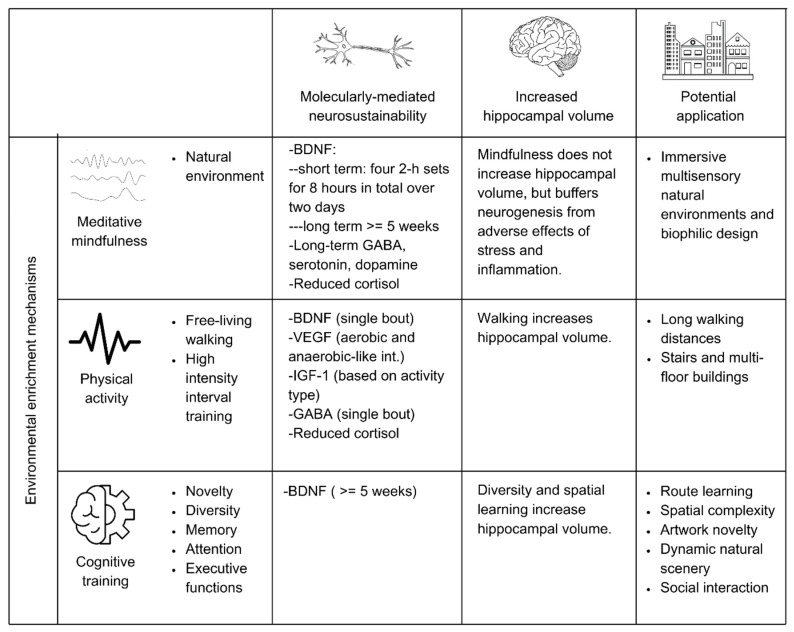
Environmental enrichment framework for hippocampal neurosustainability in humans.

**Figure 6:**
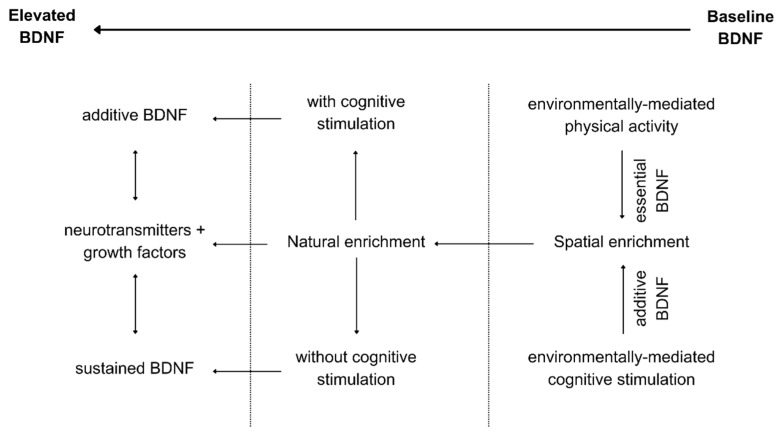
Mechanisms of BDNF increase for neurosustainability.

Hence, the challenges regarding the modern built environment’s promotion of neurosustainability through the regulation of the hippocampal neurogenesis process via the deconstruction of environmental enrichment variables [6] can be feasibly assessed using the integrative BDNF-mediated model developed in this paper, as well as through the interactions illustrated in Figure 5 and Figure 6. The reliance on changes in BDNF solely or in conjunction with other growth factors, neurotransmitters, or structural changes in the human brain can help isolate variables in order to test their immediate and long-term effects.

This model’s practical implications would greatly help to maximize the effect of environmental affordance for physical activity, which aims to stimulate sustainable BDNF release by reaching metabolic equivalents through the built environment [25]. The model developed in this paper explains that physical activity is not the only promoter for stimulating BDNF, but should be integrated with the slow stimulation of cognitive stimulation, arguably through the utilization of spatial complexity and novelty, which have been proven to increase neurogenesis in rodents as a form of cognitive stimulation [139]. More research is still needed to experiment with adding spatial complexity and novelty into the built environment. To date, in our opinion, only navigational complexity for humans is similar to that for rodents [145,156], but inducing novelty in the human context is still a challenge. Integrating both mechanisms can be highly effective in the human-built environment. However, it is also essential to improve the quality of the built environment itself to avoid inducing stress or inflammation that may interfere with the stimulation of BDNF or minimize its optimization. For instance, natural environments are proven to cause salutogenic effects, as evidenced in the hippocampal subiculum and amygdala, but not the built environment [129,130], suggesting that future research may experiment with biophilic design integration or residential greenness to reach homeostasis. Collectively, the built environment can guarantee sustainable regulation of BDNF through stimulation of physical activity, complexity, and salutogenic effects.

With the immediate and short-term changes in BDNF before it is consumed for neuroplasticity, interdisciplinary efforts are needed to take this constructive deconstruction of the mechanisms into a reconstructive built environment for the human brain. Environmental neuroscience, molecular sciences, psychiatry, public health, and neuroarchitecture researchers can collaboratively enrich the built environment with a well-informed strategy. Those strategies can be highly insightful for urban planners, architects, and public health policymakers keen on promoting brain health, sustained cognitive performance with aging, and improved mental health through the counteractive effect of neurogenesis on several mood disorders.

## 3. Conclusions

This paper proposes a novel, three-way, BDNF-interactive model that maps the hierarchical interactions between the adult hippocampal neurogenesis (AHN) stage, environmental enrichment mechanisms, and underlying molecular mechanisms toward neurosustainability through potential means of environmental enrichment in the built environment. The model facilitates future research on the affordance of physical activity, cognitive stimulation, and mindfulness through the built environment to promote hippocampal neurosustainability and mental health in humans. It enables the isolation of the effects of specific environmental factors to better inform environmental enrichment for humans by looking into the immediate and short-term effects on BDNF independently or in conjunction with each other, due to their integrative nature. This model bridges a critical gap by elucidating pathways from the environment to hippocampal function and neurosustainability through precise molecular targets like BDNF. This understanding of how to actively enrich environments to sustainably support hippocampal neurogenesis holds more significant implications for improving mental health. Future research on environmental affordance for physical activity, navigation and spatial complexity, and biophilic design can further inform environmental enrichment through the BDNF mechanisms identified in this paper.

## Data Availability

No new data were created or analyzed in this study. Data sharing is not applicable to this article.
